# Evaluation of Light Cementitious Matrix with Composite Textile Reinforcement from Garment Waste

**DOI:** 10.3390/ma16020733

**Published:** 2023-01-11

**Authors:** Kátia Jocasta Ortiz Grings, Francisco Roger Carneiro Ribeiro, Davi Vaz André Junior, Afonso Rangel Garcez de Azevedo, Marlova Piva Kulakowski

**Affiliations:** 1 Civil Engineering Graduate Program, University of Vale do Rio dos Sinos (UNISINOS), São Leopoldo 93022-750, RS, Brazil; 2 Civil Engineering Graduate Program, Federal University of Rio Grande do Sul (UFRGS), Porto Alegre 90035-190, RS, Brazil; 3 Advanced Materials Laboratory (LAMAV), State University of Norte Fluminense Darcy Ribeiro (UENF), Avenida Alberto Lamego 2000, Campos dos Goytacazes 28013-602, RJ, Brazil; 4 Civil Engineering Laboratory (LECIV), State University of Norte Fluminense Darcy Ribeiro (UENF), Avenida Alberto Lamego 2000, Campos dos Goytacazes 28013-602, RJ, Brazil

**Keywords:** lightweight matrices, vermiculite aggregate, textile cement composites, textile treatment, polyester garment waste

## Abstract

The use of recycled waste has been the focus of several studies due to its potential to allow a more sustainable use of construction materials and minimize improper waste disposal in landfills or incinerators. More specifically, garment textile waste has been examined as internal reinforcement of cementitious matrices to increase the deformability and control fissure formation. In this study, polyester textiles are analyzed and incorporated in cementitious composites in order to evaluate their mechanical properties. Results show that significant improvements in mechanical properties of composites are obtained depending on the impregnation treatment applied to the textile waste. In the direct tensile stress test, the waste impregnation with styrene butadiene polymer plus silica fume improved 35.95% in the weft direction and 9.33% in the warp direction. Maximum stress increased 53.57% and 64.48% for composites with styrene–butadiene rubber impregnation and styrene–butadiene rubber plus silica fume impregnation, respectively, when compared to the unreinforced composite. The flexural tensile strength of composites impregnated reinforcements with styrene–butadiene rubber and styrene–butadiene rubber plus silica fume presented increases in strength by 92.10% and 94.73%, respectively, when compared to the unreinforced sample. The impact test confirmed that styrene–butadiene rubber plus silica fume impregnation produced greater tenacity of the composite. In the microstructure, it is confirmed that the impregnated textile reinforcement resulted in composites with greater adhesion between the fabric and the cementitious matrix. Thus, light textile waste is concluded to be a viable construction material for non-structural elements.

## 1. Introduction

Worldwide production of textile fibers doubled in the last few decades and surpassed 107 million tons in 2018 [1]. According to the 2020 Preferred Fiber and Materials Market Report [2], synthetic fibers dominated the market, with polyester accounting for 50% of total global production with approximately 57 million tons per year. In comparison, cotton was the second most used textile fiber and amounted to 26 million tons, less than half of polyester production [1]. The yearly production of fibers for clothing textiles was estimated at 53 million tons, of which only 12% were later recycled and 73% eventually sent to landfills or incinerators [3].

Current management systems have shown to be inadequate to deal with the large amount of textile waste generated daily. This resulted in a significant environmental issue and a lack of tracking data regarding the amount generated of each type of textile waste [4]. Textile wastes could be post-consumption garments discarded by consumers after use or post-industrial generated during the manufacturing process [5]. The manufacturing of garments involved optimized cuts which resulted in waste patches of different sizes and shapes. Additionally, distinct compositions and textures could occur due to the structure, thickness, and arrangement of fibers. This presented a considerable challenge in determining applications for textile wastes [6].

A possible solution to this environmental issue would be repurposing waste into coproducts, which could also contribute to energy savings and conservation [7]. However, challenges regarding adequate performance and economic viability must be overcome [2,6]. From a technical viability, recycling textile waste required finding alternatives and materials that could accept the incorporation of waste. Textile waste was examined in other studies [8] in several applications such as thermal insulation in buildings [7,9], sound proofing [10,11], and light bricks [12,13].

Short fibers from polyester textile waste [14] and polyester/cotton mixtures [15] were evaluated as sustainable reinforcement in structural cementitious composites with low performance requirements. Most of these studies dispersed short fibers randomly in the matrix. This was a necessity since fiber waste usually consisted of different sizes and shapes and required processing into a particulate before use [16]. As a result, these techniques produced limited improvement in concrete strength and tenacity due to the short dimensions of the particulate (in the order of 1 to 10 mm, up to a few cm) and the small volume addition to the composite (usually 0.2 to 2% in volume) [17,18].

In contrast to short fibers dispersed randomly in the matrix, longer fiber in textile and nonwoven fabric reinforcements could bring more significant improvements to the mechanical properties of composites. The lengths associated with longer fibers increased contact with the cement paste while uniform distribution and multidirectional reinforcement of textiles (in both weave directions) could lead to the formation of multiple micro-cracks and improvements in mechanical properties such as tensile and flexing strengths and impact testing [18,19,20,21]. Some examples were studies considering sustainable textile or unconventional reinforcements such as sisal fibers [21,22], flax-based cellulose textiles [23,24], jute fibers [25,26], nonwoven flax cloth [19,27], and nonwoven cloth from polyester and cotton waste from the garment and textile industries [28]. The use of textile waste could be further aided with techniques that impregnate it with resin to improve textile-matrix bonding. These techniques were previously successfully tested in conventional textiles [25]. These studies demonstrated that cementitious composites with textile reinforcement had improved tensile and flexing performance and tension-induced hardening despite the fibers themselves having a low modulus of elasticity. However, there is a need for further studies in the use of polyester nonwoven cloth as reinforcement in light matrices.

Thus, this study was conducted to evaluate the use of polyester waste from the garment industry as reinforcement in cementitious composites with non-structural uses such as furniture and light partitions. Benefits of the use of this reinforcement would be increased ductility and cracking control.

## 2. Materials and Methods

### 2.1. Characterization of Materials

The concrete composite matrix was elaborated in accordance with standard NBR 16697 [29] and made use of Portland cement CP II-F-40. This type of cement had filler with higher fineness modulus and was recommended for applications requiring rapid demolding or in the production of pre-fabricated elements. Textile waste was treated with silica fume (SF) obtained from foundries processing metallic and iron silicates. This compound was selected to improve the mortar–fiber transition zone since it was extremely fine, reactive, and contained amorphous silica. The chemical and physical characterization of the materials are shown in Table 1.

In order to obtain a concrete composite with low specific mass, expanded vermiculite (EV) was used as light aggregate in the mix ratio. Physical characterization of EV yielded a bulk density of 0.14 g/cm^3^, specific mass of 1.14 g/cm^3^, and water absorption of 231%. According to standard NBR 11355 [30], this EV was classified as superfine while in standard NBR 7211 [31], it was classified as fine.

A polycarboxylate superplasticizer was used in order to produce a cementitious matrix with appropriate rheology for light aggregate dispersal without exudation or segregation. A viscosity-modifying additive was also used to increase concrete viscosity in order to control slump and prevent segregation. Finally, an auxiliary air-detrainer additive was incorporated to remove foaming and trapped air bubbles from the composite in order to prevent the excessive formation of pores on the surface.

### 2.2. Matrix Design

The water/cement (w/c) ratio of the mixture was fixed at 0.40 and the optimum saturation point of each chemical additive determined from this starting parameter. The effect of the additives over time was checked with Kantro mini slump tests at 0, 5, and 15 min after mixing. Additive contents tested were of 0, 0.1, 0.2, 0.3, 0.4, and 0.5%. Based on the results, optimum contents of 0.30% were determined for the superplasticizer and 0.20% for the viscosity-modifying additive.

Once the superplasticizer and viscosity-modifying additive contents were determined, mini slump-flow tests were performed to determine the optimum content of light aggregate for a self-consolidating concrete in accordance with standard NBR 15823-2 [32]. Design parameters were an SF2 slump class adequate for non-reinforced or low-reinforcement structures with short slumps, a VS1 class for t500 apparent plastic viscosity, and an IEV class 0 of visual stability index. Mixtures with EV contents of 55, 60, 65, 70, and 75% with respect to the mass of cement were tested. Prior to mixing, EV was pre-saturated for 30 min with a water content determined from the light aggregate absorption test. This was necessary since EV absorbed water during mixing and could reduce both workability and slump of the resulting composite. The density of all mixtures was also measured with the procedures of standard NBR 13278 [33] and determined to be below 2000 kg/m^3^. The densities matched the criteria for light mixtures stipulated by standard NBR 8953 [34]. For this part of the study, the mixtures were produced in a 5 L mixer under controlled temperature and humidity levels and no air-detrainer additive. The optimum EV content was thus determined to be 55%.

The final step to obtain the optimum mix ratio of the concrete was to determine the air-detrainer additive content. Mini-slump tests were performed on mixtures with ideal EV, superplasticizer and viscosity-modifying additive, and varying air-detrainer content. Contents tested varied from 0 to 1.00% in 0.10% increments with respect to the mass of cement. Similar to previous steps, the density of each mixture was measured with the procedures of standard NBR 13278 [33] and all values in the hardened state were below 2000 kg/m^3^. Cylindrical test bodies (50 mm × 100 mm) were produced with each mixture in order to perform a visual evaluation of surface porosity. The ideal air-detrainer content was thus determined to be 0.20%.

The mechanical behavior of the resulting light cementitious matrix was evaluated with compression strength tests at 3 days, 7 days, and 91 days of curing. Tests were conducted on cylindrical test bodies in accordance with the procedures of standard NBR 7215 [35] and the results were of 0.44, 1.01, and 6.98 MPa, respectively.

### 2.3. Polyester Textile Waste

**Figure 1:**
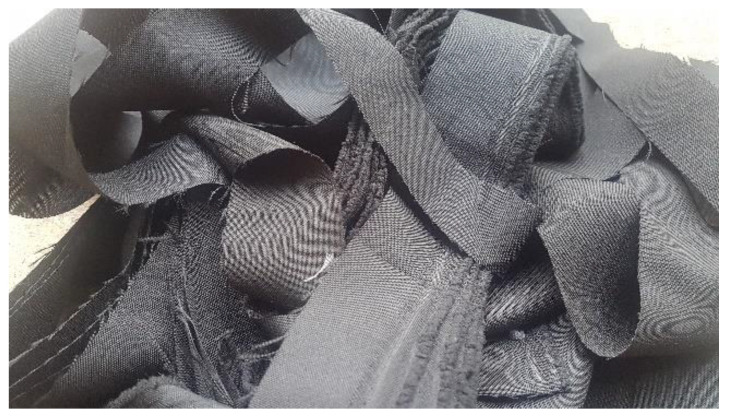
Polyester waste selected for this study (post-triage).

Textile wastes were leftovers from clothing molds and sourced from a garment industry. A triage was performed to select patches with similar specifications: type of textile, color, weave direction, and dimensions in order to reduce variability in the results of this study. The selected waste consisted of polyester textile, as seen in Figure 1, with a specific mass of 1.38 g/cm^3^ and its threads have thickness in the order of 200 μm (0.2 mm). The textile is manufactured from two strands weaved perpendicular to each other in alternating over/under order. The strands arranged along the length are called warps, while the strands in the transverse direction of the fabric are called weft, identified by the selvage direction.

Following the procedure of other studies on concrete with textile fibers [25,26], styrene–butadiene rubber (SBR) was impregnated into the polyester waste in order to improve fiber/paste interface adhesiveness. The SBR had an alkaline pH and specific mass of 1.050 g/cm^3^.

For this study, the polyester textile waste was impregnated with two techniques: SBR only or a mixture of SBR + SF. Impregnation of the textile waste sought to improve interface adhesiveness between fibers and the matrix and fill gaps in between filaments with silica particles. Impregnation was performed by immersion of the textile in either an SBR solution or an SBR + SF solution of 1:1 mass ratio for 50 min, which was observed to be the length of time for maximum absorption. The polyester waste was cut in strips in pre-defined directions (along or warp or weft) into sizes specific for each type of test. Strips of 50 mm × 200 mm were cut for isolated direct tensile tests applied only to the textile, while strips 25 mm wide but 10 mm shorter than the length of the reinforced concrete test bodies produced for mechanical tests. After soaking, the strips were stretched and dried for 24 h.

In order to characterize the chemical composition of the textile, a sample of untreated impregnated polyester fabric was analyzed by Fourier Transform Infrared Spectroscopy (FTIR).

Textile morphology and weave were examined in untreated samples (without impregnation) in a model Zeiss Visioner 1 apparatus (USA). The treated textiles with SBR or SBR + SF impregnation were gold-metallized and analyzed with Scanning Electronic Microscopy (SEM) in a model EVO MA 15 apparatus (Urbana, IL, USA).

Mechanical behaviors of untreated and impregnated samples were evaluated with direct tensile strength tests applied solely to the textiles. Testing was conducted on flat samples both along warp and weft directions. Testing procedure followed the recommendations of standard NBR ISO 13934-1 [36] in an EMIC-INSTRON universal press with 5 kN load and displacement rate of 10 mm/min.

### 2.4. Composite Production and Analysis

**Figure 2:**
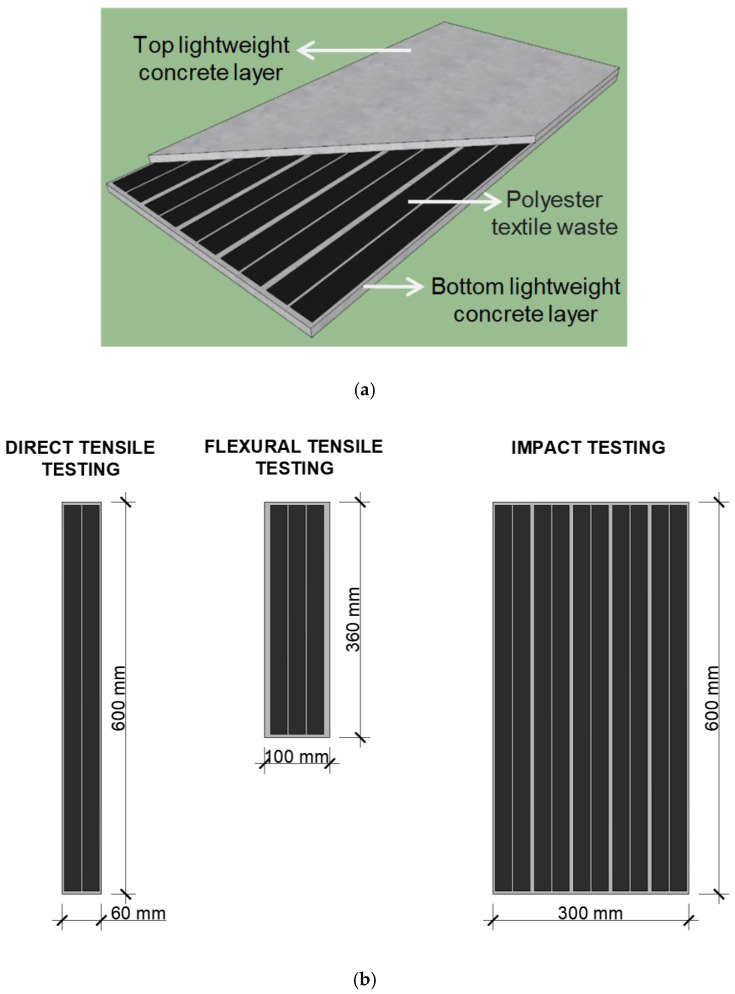
(**a**) 3D schematic of the layers of the composite; (**b**) dimensions and placement of textile reinforcements and test body sizes for each mechanical performance test.

**Figure 3:**
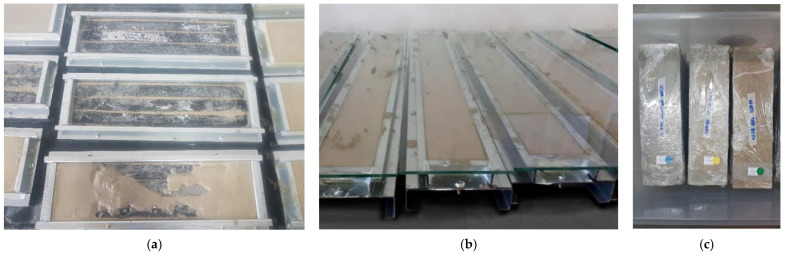
(**a**) The preparation of samples: bottom lightweight concrete layer according to the level marked on the metallic form, positioning of polyester strips, and filling of top lightweight concrete layer; (**b**) closed form with glass; (**c**) test bodies demolded after 72 h, wrapped in plastic wrap, and stored in closed plastic containers with a layer of water at the bottom.

Concrete composites were produced in a vertical-axis planetary cement mixer and poured on metallic molds of test bodies specific for each test. Pouring consisted of an initial layer of light concrete, polyester strips, and a covering of light concrete. Thus, the test body was composed of two external layers of light concrete and polyester textile strips in the center. The molds had markings to ensure that both concrete layers would be poured to the same thickness. After pouring, the molds were covered with a glass sheet and no vibrating compaction was applied since the concrete was self-consolidating. Figure 2a shows a 3D schematic of the layers of the composite while Figure 2b shows the dimensions and placement of each textile reinforcement strip and dimensions of the test bodies used for each mechanical performance test. The direct tensile testing, the flexural tensile, and the impact testing used 2, 3, and 10 fabric strips, respectively. The dimension of the fabrics for each test were 25 × 590 mm, 25 × 350 mm, and 25 × 590 mm. Figure 3a shows the preparation of samples for testing while Figure 3b the samples covered with a glass after molding.

Test bodies were demolded after 72 h, wrapped in plastic wrap, and stored in closed plastic containers with a layer of water at the bottom according to Figure 3c. The test bodies remained stored in the containers for 24 h prior to testing, at which point the water was drained and plastic wrap removed. Direct tensile tests were performed on four test bodies measuring 600 × 60 × 18 mm cured for 91 days. Testing was conducted with an EMIC/Instron universal press (model EMIC CCE300KN, Paraná, Brazil) with a controlled loading rate of 1.0 mm/min as recommended by standard TC 232-TDT (RILEM, 2016). Cracks were visually identified from photographic images and measurements of width and cracked area performed in AutoCad 2018 software.

Flexural strength tests were also conducted with the same equipment on test bodies measuring 360 × 100 × 18 mm cured for 91 days. In this case, a 4-point test was performed with a controlled loading of 2.0 mm/min following standard ASTM C947-03 [37] and recommendations of Vlach et al. [38].

Impact testing procedures were adapted from standard NBR 11675 [39]. The test body 600 × 300 × 18 mm was placed horizontally on a wooden frame measuring 600 × 300 mm. A total of four samples were tested. The test body face was then subjected to impacts at random locations from a steel sphere 5 cm in diameter and 500 g of mass. Consistent impact heights were obtained by positioning the sphere with a PVC pipe so that its center of mass would be 50 cm or 100 cm above the test body, corresponding to impact energies of 2.5 J or 5.0 J, respectively. Each concrete composite test body was subjected to up to 10 impacts from each height. Divot depths were measured and cracks visually identified from photographic images taken at a distance of 50 cm. Fissure identification was conducted on AutoCad 2018 software and measured length, width and fissured area.

Microstructural analysis of the concrete composites with textile reinforcement were conducted with SEM imaging on gold-metallized samples measuring 5 × 5 × 5 mm. Prior to analysis, samples were placed in isopropyl alcohol for 15 min and dried in an oven at 40 °C to stop the hydration process of cement.

## 3. Results and Discussion

### 3.1. Textile Reinforcement Characterization

**Figure 4:**
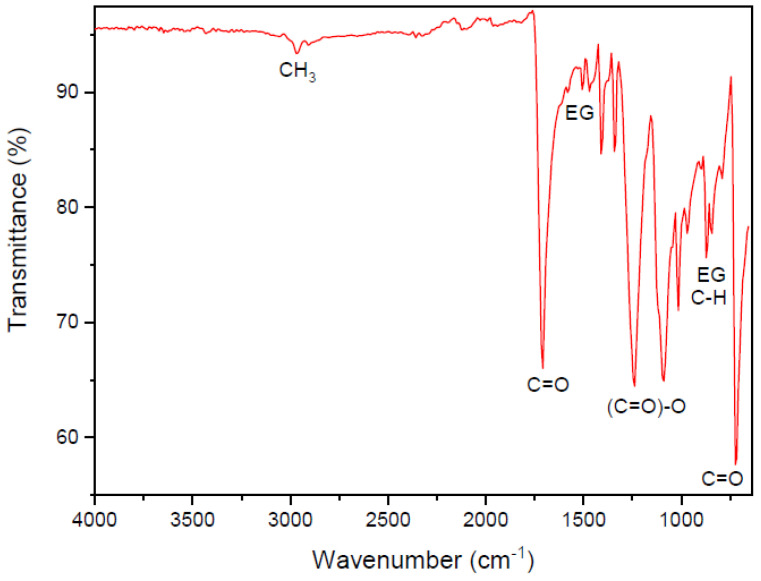
FTIR of the polyester textile.

Figure 4 shows the FTIR spectrum of the polyester textile. The sample shows typical polyester polymer deformations in the regions of 2900 cm^−1^ for CH_3_; 1715 and 730 cm^−1^ for the C=O group; 1460 and 977 cm^−1^ for the ethylene glycol (EG); and 1250 cm^−1^ for the (C=O)-O group.

**Figure 5:**
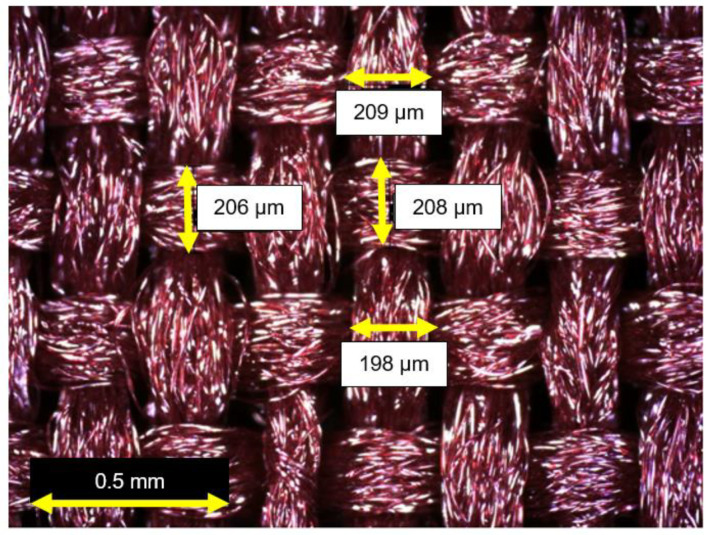
Polyrester textile morphology.

The polyester waste was classified as a planar 2D textile manufactured from two strands weaved perpendicular to each other in alternating over/under order. Each strand was composed of long filament fibers grouped in bundles as shown in Figure 5. When grouped in bundles, the fibers were considered as multifilaments.

**Figure 6:**
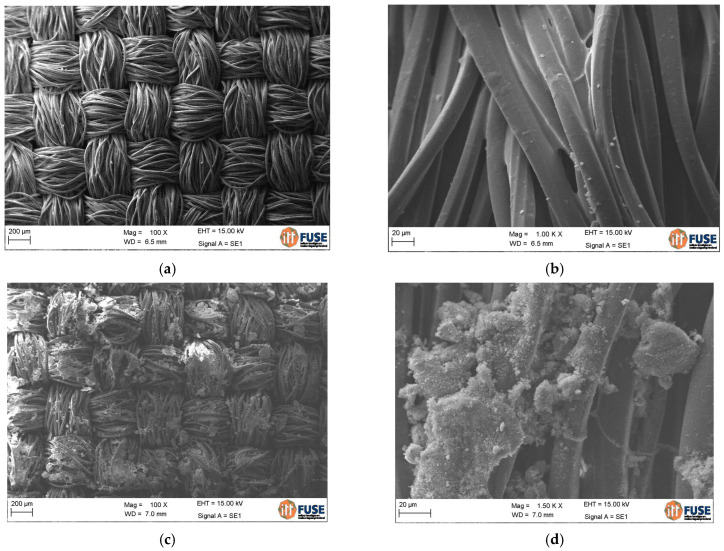
SEM images of: (**a**) untreated polyester textile; (**b**) polyester impregnated with SBR; (**c**,**d**) polyester impregnated with SBR + SF under different magnifications.

The textile waste had a thickness of around 200 μm (0.2 mm), which was used as a base value to determine the cross-sectional area and categorize tensile strength of the material. This thickness did not increase when the textile was impregnated but improved multifilament grouping was observed as shown in Figure 6. This result was also reported by Hegger and Voss [40].

**Figure 7:**
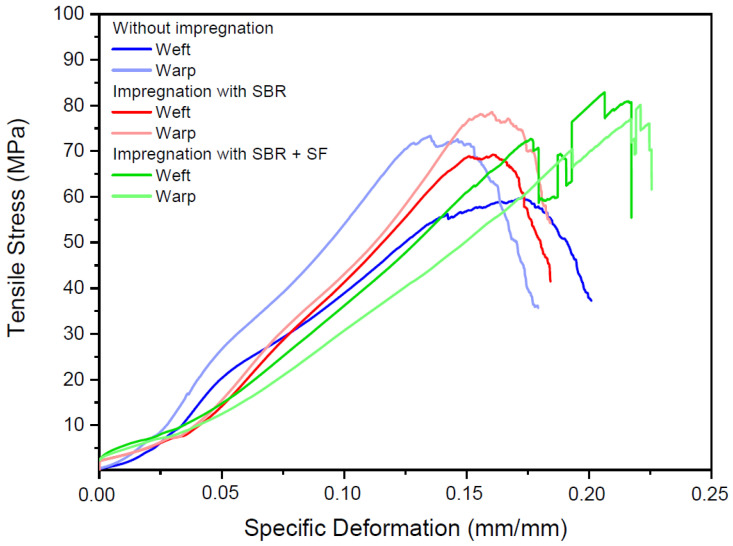
Stress–deformation curves along the weft and warp of polyester samples without treatment and with impregnation.

Figure 7 presents the mechanical behavior of the textile waste when subjected to direct tensile stresses along the weft and warp of the fabric. There was an observable difference in the stress–deformation curves of polyester samples without treatment and with impregnation.

There are three types of stress–strain behavior of polymeric materials: fragile polymer that undergoes fracture while elastically deforming and occurs without observing the initial deformation of the material; ductile polymer, whose initial deformation is elastic, followed by yielding and a plastic deformation region; and finally fully elastic deformation whose large deformations (even under small stress levels) are recoverable [41,42]. Compared to impregnated samples, untreated polyester had fracture types clearly characteristic of ductile materials with significant deformation until rupture. Samples impregnated only with SBR presented a similar behavior, with an extensive initial elastic deformation, followed by flowing and plastic deformation zones. On the other hand, polyester impregnated with SBR + SF presented a different behavior: plastic deformation did not occur past the flowing zone and rupture occurred abruptly. This indicated that the SBR + SF sample could be classified as a fragile material.

**Figure 8:**
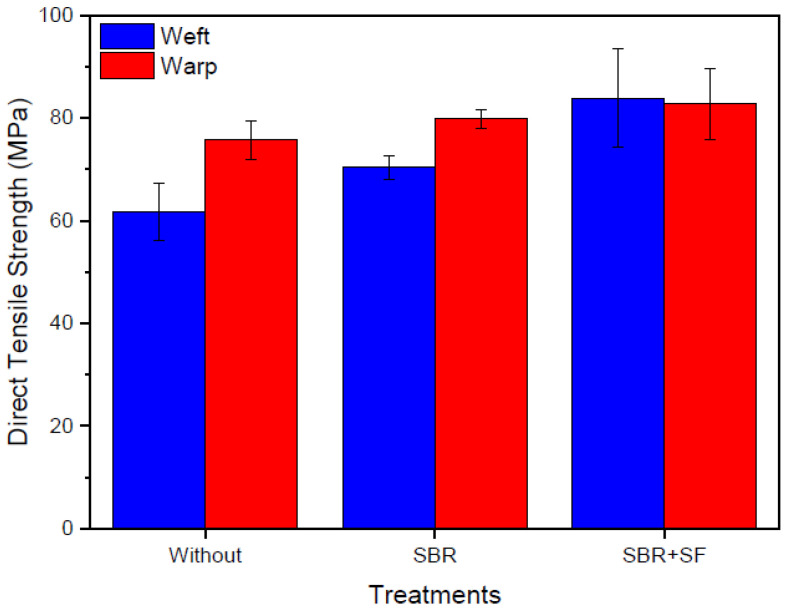
Direct tensile strengths along weft and warp for polyester samples with and without impregnation.

Figure 8 presents average maximum tensile strengths of the textile samples. Untreated polyester had average strength of 61.67 MPa along the weft and 75.66 MPa along the warp. These values were within the expected range of 48.30 to 72.40 MPa reported by Callister [41]. Treated polyester presented gains from impregnation: along the weft, SBR impregnated samples had an increase of 14.03% while SBR + SF impregnated samples had an increase of 35.95%. Along the warp, increases were of 5.47% and 9.33% for the SBR and SBR + SF samples, respectively. However, the variability of the results suggested a caveat with regard to improvements in mechanical behavior. Still, references suggested clear benefits of impregnation. Fidelis et al. [26] observed increases of up to 24% on jute textile samples impregnated with SBR when compared to untreated samples. Moreover, Triantafillou [43] described improvements in internal filament grouping when compared to untreated filaments, which led to an increase in strength.

Overall, average tensile strengths had better performances both for untreated samples and SBR impregnated samples. The relative increases along warp with respect to weft values were of 22.69% and 13.48% for untreated and SBR impregnated samples, respectively. Haik et al. [44] evaluated the mechanical behavior of planar textiles with respect to tension and observed better performance along warp than weft. This result was considered to be an effect of filaments weaved across the weft creating increased friction, which did not occur along the more isolated sliding filaments of the weft. However, for textiles impregnated with SBR + SF, tensile strengths along warp and weft were equivalent, which was a positive result considering the industrial nature of the textile waste. Often such waste samples contained a variety of shapes and sizes which could make impossible the exact identification of weft and warp directions.

### 3.2. Analysis of the Composites

**Figure 9:**
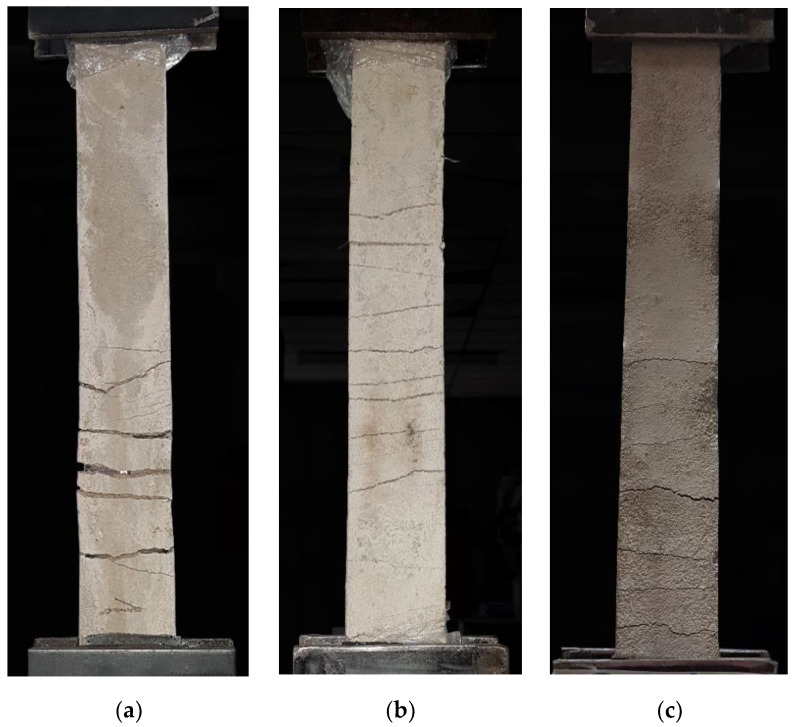
Fissure formation in test bodies with (**a**) untreated reinforcement, (**b**) SBR impregnated reinforcement, and (**c**) SBR + SF impregnated reinforcement.

As seen in Figure 9, the concrete textile composite under direct tensile stress was characterized by the formation of multiple cracks. However, as seen in Table 2, this result was coupled with positive indicators from composites with impregnated textiles whose samples presented a higher number of cracks but with smaller widths when compared to the composites with untreated textiles. The composite with untreated textiles of Figure 9a contained 5 cracks with an average crack width of 2.86 mm, the composite with SBR impregnated textiles of Figure 9b contained 11 cracks with an average crack width of 1.09 mm and the composite with SBR + SF impregnated textiles of Figure 9c contained 7 cracks with an average crack width of 0.59 mm. The cracks have a width 61.89% and 79.37% smaller than the untreated samples, respectively. As for the cracked area, the same behavior is presented, the samples with treated reinforcement have a cracking area 61.34% smaller for the composite with SBR impregnated textiles and 71.94% smaller for the composite with SBR + SF impregnated textiles than the sample with untreated reinforcement.

**Figure 10:**
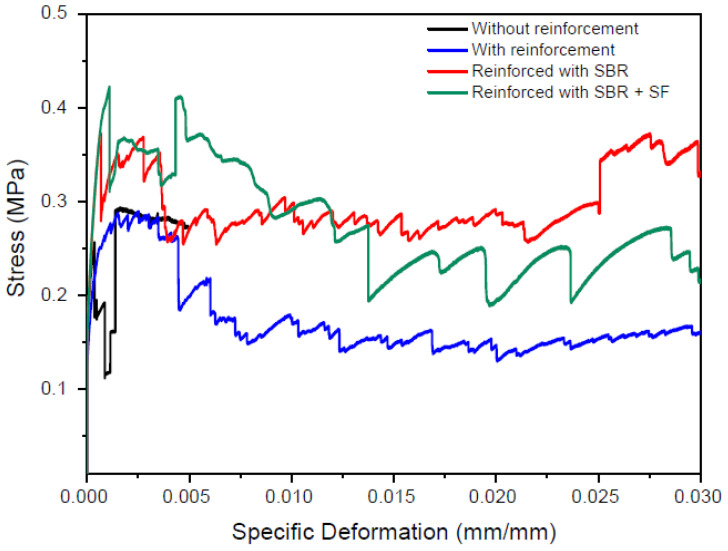
Stress vs. deformation of the concrete composites.

Table 3 shows the average results of the direct tensile testing of the samples. Figure 10 shows stress vs. deformation curves for all composites of this study: unreinforced, untreated reinforcement, SBR impregnated reinforcement, and SBR + SF impregnated reinforcement. Results show increases in maximum stress of reinforced composites compared to unreinforced ones. The average tensile strength of the textile composite and their standard deviation were, respectively, 0.28 MPa and 0.02 MPa for the unreinforced sample; 0.31 MPa and 0.13 MPa for untreated reinforcement sample; 0.43 MPa and 0.06 MPa for SBR impregnated reinforcement sample; and 0.46 MPa and 0.02 MPa for SBR + SF impregnated reinforcement sample. Furthermore, composites with treated reinforcements presented the most significant increases. Maximum stress increased 53.57% and 64.48% for composites with SBR impregnation and SBR + SF impregnation, respectively, when compared to the unreinforced composite.

The composite with untreated reinforcement presented an increase in maximum stress of 10.71% with respect to the unreinforced sample. Comparison of the performance of untreated reinforcement and impregnated reinforcement composites showed an increase of up to 48.39% in maximum stress for the SBR + SF impregnated composite. However, the standard deviation of the untreated reinforcement composite was quite elevated, which prevented definitive confirmation of its performance with respect to both unreinforced and impregnated reinforcement samples. Nili and Afroughsabet [45] also confirmed that the addition of synthetic fibers improved the tensile strength of composites. In the case of Nili and Afroughsabet [45], strength at 28 days of cementitious composites with 0.2, 0.3, and 0.5% SF content and w/c of 0.46 increased 15, 20, and 27%, respectively. Additionally, for the same composites with a w/c of 0.36, the increases in strength were of 15, 16, and 23%, respectively.

**Figure 11:**
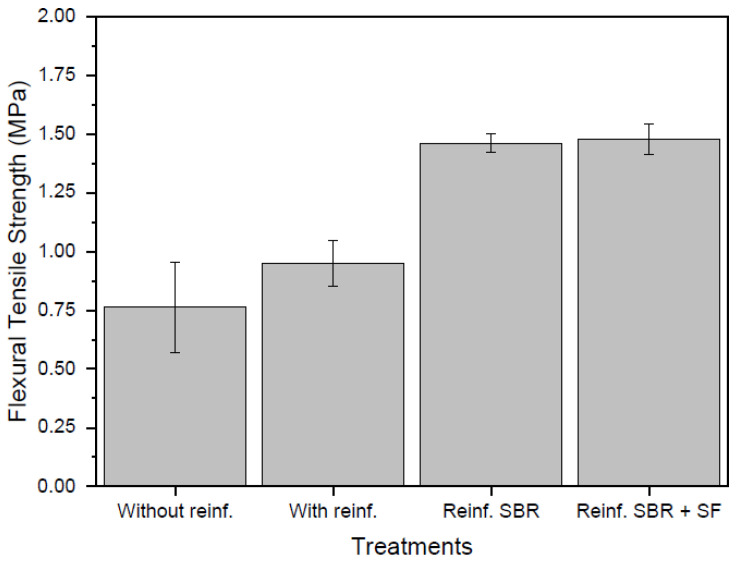
Flexural tensile strength of the composites.

Figure 11 presents average flexural tensile strength of all composites of this study. Results were within expected parameters since the composite was a light concrete for non-structural applications. Concretes with EV light aggregate were known to have a compressive strength below 7 MPa and tensile strength usually corresponded to 10% of compressive strength. The average flexural tensile strength of the textile composite and their standard deviation were, respectively, 0.76 MPa and 0.19 MPa for the unreinforced sample; 0.95 MPa and 0.10 MPa for untreated reinforcement sample; 1.46 MPa and 0.04 MPa for SBR impregnated reinforcement sample; and 1.48 MPa and 0.07 MPa for SBR + SF impregnated reinforcement sample. Composites with SBR and SBR + SF impregnated reinforcements presented increases in strength of 92.10% and 94.73%, respectively, when compared to the unreinforced sample. Additionally, comparison of these same 2 composites with the untreated reinforcement sample showed increases of 53.68% and 55.78%, respectively. In the case of the untreated reinforcement composite, a 25% increase in strength was noted with respect to the unreinforced sample. However, due to the high standard deviation of the unreinforced sample, this increase cannot be effectively ascertained from the results of this study.

Increases in flexural tensile strength for composites with synthetic fibers when compared to unreinforced samples were also reported by Nili and Afroughsabet [45]. Furthermore, the increases were even higher for samples incorporating SF and fibers. Zhou et al. [46] also reported positive results for flexural strength with the combined use of polyester and SBR in concrete. This was concluded to be the effect of improved fiber and cement paste bonding from SBR when compared to unreinforced concrete. Additionally, since polyester fibers had excellent water-resistant characteristics, it resulted in both improved adhesiveness and protection from degradation.

**Figure 12:**
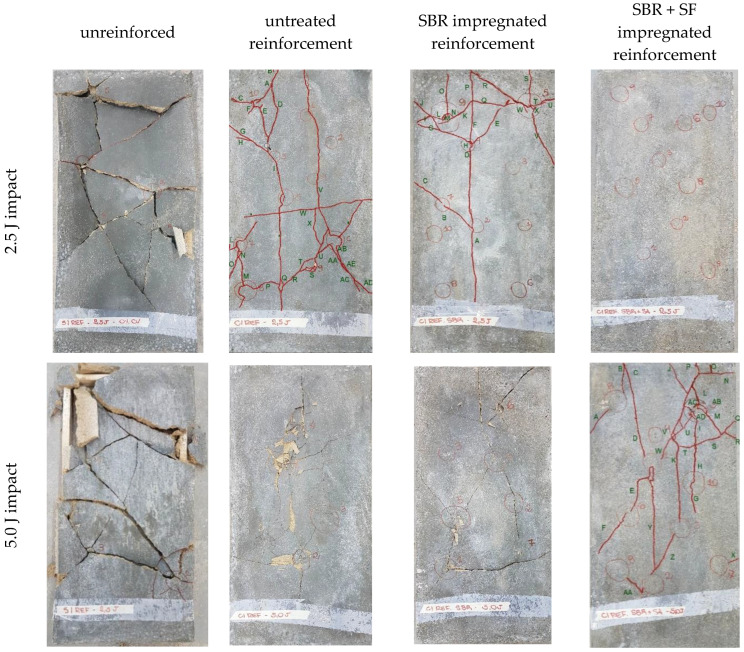
Visual surface appearance of test bodies for all composites subjected to impact testing.

Figure 12 shows the visual surface appearance of the test specimens used in this study after impacting testing while Table 2 shows the average depths, divot diameter, and fissured areas produced from the impacts.

In Table 4, no depth, diameter, or fissured area was presented for the unreinforced composite with the 2.5 J impact because the test bodies ruptured after 5 impacts. This result demonstrated the efficiency of textile reinforcement in increasing impact strength of the composites. It should be noted that cracks and divots only appeared in the reinforced composites after 10 impacts of 2.5 J. For the reinforcement composites subjected to 2.5 J impacts, the untreated sample presented more cracks than impregnated ones. More specifically, the untreated composite had 46.49% larger fissured area than the SBR impregnated sample. Furthermore, visual inspection determined that the best performing composite was the one containing textiles impregnated with SBR + SF since no cracks were visible at the image distance of 50 cm.

Results for the 5.0 J impact test were only registered for the composite with SBR + SF impregnated reinforcement since all other samples ruptured completely after 5 impacts. Thus, in general, it was confirmed that SBR + SF impregnation produced greater tenacity of the composite for both 2.5 J and 5.0 J impacts. A possible explanation for this result would be that, since SF is a pozzolan, its addition strengthened the transition zone and decreased initial fissure formation. Nili and Afroughsabet [45] noted that the addition of SF increased the number of impacts necessary to induce the first fissure by 6 to 8.5 times and this was evidence of an increase in impact resistance of these concrete composites.

**Figure 13:**
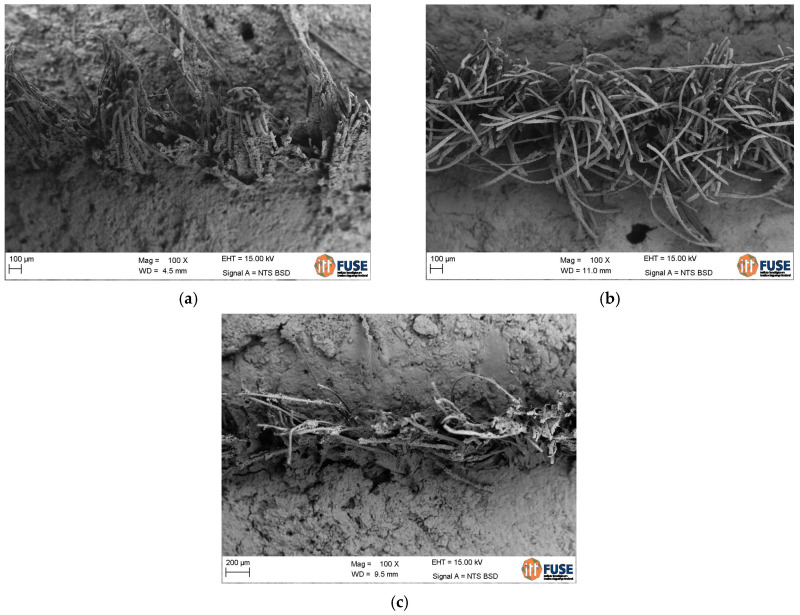
Microphotography of composites with textile reinforcement: (**a**) untreated, (**b**) SBR impregnated textile, and (**c**) SBR + SF impregnated textile.

Figure 13 shows microphotography of the microstructure of the reinforced composites and typical morphology of the interface zone between multifilament yarns in the matrix. For untreated fibers, the matrix does not seem well incorporated into the fibers in the composite matrix, while for treated yarn, it can be seen that due to the impregnation, the polyester fiber textile is better incorporated into the matrix. As seen in Figure 13c, the composite with SBR + SF impregnated reinforcement had a few void spaces near the textile, but the cementitious matrix was able to penetrate in between filaments with greater efficiency. In the case of the composite with SBR impregnated reinforcement of Figure 13b, the filaments ended up well adhered to the matrix.

For treated yarns, it can be seen that the fibers are covered with a thick layer of polymeric material. The polymer impregnated yarns are in good condition and the hydration products are grown together with the impregnation layer.

The same behavior was described by other authors [25,26] for uncoated fibers in the reinforced matrix. It can be seen that Portlandite crystals have grown between the brittle fibers at the interface, while for coated yarn, the fibers are protected against the alkali by the impregnation, but they break because the bond is very strong and the fibers cannot freely deform (lack of free length).

Thus, it was determined that impregnated reinforcement resulted in composites with improved adhesiveness between the textile and the cementitious matrix. Impregnation tended to produce a stronger bond between polyester fibers and cement paste. This attenuated issues due to macro and micro defects in the transition interface zone between the textile and cementitious matrix, leading to increased tenacity and resistance to fissuring. Evaluating the results, it can be seen that changes in the fiber matrix interface can modify the mechanical behavior of the composite, influencing the strength and pullout work. The composites with SBR + SF impregnated reinforcement had superior results since their stable interface could be observed.

## 4. Conclusions

The main conclusions drawn from this study were:

- The polyester used as reinforcement was a planar 2-D textile manufactured from two filaments weaved perpendicular to each other (0°/90°) with alternating over/under crossing.
- Maximum direct tensile strength of the isolated textile was within expected values for polyester. However, the high variability of results produced a caveat with respect to improvements in mechanical performance of both untreated and impregnated textiles.
- Average maximum stress in the warp direction had more satisfactory performance for both untreated and SBR impregnated textiles. In the case of SBR + SF impregnated textiles, the maximum stresses in the warp and weft directions were equivalent. This was considered to be a positive result which would allow the use of textile waste with difficult identification of weave direction.
- Characterization of the composites confirmed improvements with the use of impregnated textile reinforcement. Direct tensile tests demonstrated that the composite with SBR impregnated reinforcement had a larger number of cracks with smaller widths when compared to composites with untreated reinforcement. Measured maximum direct tensile stress increased up to 53.57% and 64.28% for composites with SBR and SBR + SF impregnated reinforcement, respectively, when compared to composites with no reinforcement.
- Flexural tensile strength presented increases of 92.10% and 94.73% for composites with SBR and SBR + SF impregnated reinforcement, respectively, when compared to unreinforced composites. Comparison of the same two impregnated reinforcement composites with untreated reinforced samples yielded increases in strength of 53.68% and 55.78%, respectively.
- Impact testing determined that, in general, reinforced composites had increased tenacity. In particular, the composite with reinforcement impregnated with SBR + SF presented the highest tenacity both with 2.5 J and 5.0 J impacts, which further confirmed the advantages of impregnated textiles.
- Microstructural analysis of the composites with impregnated reinforcement confirmed improved adhesiveness between the textile and the cementitious matrix. Impregnation strengthened the transition zone between the textile and cementitious matrix, thus increasing tenacity and resistance to fissuring.

Finally, results confirmed the possibility of producing light cementitious composites for non-structural applications with textile reinforcement from recycled long fibers from the garment industry. The use of this textile waste in construction should be promoted as a technique to minimize the impact of civil construction on natural resources.

## Figures and Tables

**Table 1:** Chemical and physical characteristics of the materials.

Characteristic	Chemical Compound and Properties		Cement	Silica Fume
Chemical (% wt.)	SiO_2_		20.16	91.42
	Al_2_O_3_		4.78	1.96
	Fe_2_O_3_		2.52	0.13
	CaO		60.76	0.53
	Na_2_O		0.38	-
	TiO_2_		0.24	-
	K_2_O		0.86	2.72
	MgO		1.51	-
	P_2_O_5_		0.12	-
	SO_3_		2.75	1.34
	Cr_2_O_3_		-	-
	MnO		0.04	-
	SrO		0.25	-
	ZnO		-	-
	LOI		5.63	1.90
Physical	Specific mass (g/cm^3^)		2.99	2.21
	Specific surface (m^2^/g)		1.55	17.77
	Laser granulometry (wet measurement)	D10 (µm)	5.91	1.73
		D50 (µm)	13.59	6.35
		D95 (µm)	34.80	14.66
		Dave (µm)	15.13	6.70

**Table 2:** Average width and fissured area for direct tensile testing of the composite.

Composite	Width (mm)	Fissured Area (mm^2^)	Fissure Content (%)
unreinforced	-	-	-
untreated reinforcement	2.86	1322.00	3.67
SBR impregnated reinforcement	1.09	811.00	2.25
SBR + SF impregnated reinforcement	0.59	371.00	1.03

**Table 3:** Results of the direct tensile testing of the composite (values in the parentheses correspond to the standard deviation).

Sample	Stress 1st Crack (MPa)	Deformation 1st Crack (%)	Maximum Tensile Force (MPa)	Maximum Deformation(%)
Unreinforced	0.26 (0.03)	0.04	0.28 (0.02)	0.07 (0.03)
Untreated	0.22 (0.02)	0.04	0.31 (0.13)	0.26 (0.15)
SBR	0.37 (0.10)	0.06	0.43 (0.06)	1.03 (1.49)
SBR + SF	0.42 (0.04)	0.10	0.46 (0.02)	0.20 (0.13)

**Table 4:** Average depth, divot diameters, and fissured area from impact testing on all composites.

Composite		Depth (mm)	Diameter (mm)	Fissured Area (mm^2^)	Fissure Content (%)
2.5 J	unreinforced	-	-	-	-
	untreated reinforcement	2.98	16.54	601.82	0.33
	SBR impregnated reinforcement	1.10	14.02	341.72	0.19
	SBR + SF impregnated reinforcement	1.36	15.65	11.86	0.007
5.0 J	SBR + SF impregnated reinforcement	3.55	21.26	1658.54	0.92

## Data Availability

Data sharing not applicable.
